# A Cost-Effectiveness Analysis of First Trimester Non-Invasive Prenatal Screening for Fetal Trisomies in the United States

**DOI:** 10.1371/journal.pone.0131402

**Published:** 2015-07-02

**Authors:** Brandon S. Walker, Richard E. Nelson, Brian R. Jackson, David G. Grenache, Edward R. Ashwood, Robert L. Schmidt

**Affiliations:** 1 ARUP Laboratories, Salt Lake City, Utah, United States of America; 2 Department of Internal Medicine, Division of Epidemiology, University of Utah, Salt Lake City, Utah, United States of America; 3 Department of Pathology and ARUP Laboratories, Salt Lake City, Utah, United States of America; 4 Department of Pathology and ARUP Laboratories, University of Utah, Salt Lake City, Utah, United States of America; University Hospital Basel, SWITZERLAND

## Abstract

**Background:**

Non-invasive prenatal testing (NIPT) is a relatively new technology for diagnosis of fetal aneuploidies. NIPT is more accurate than conventional maternal serum screening (MSS) but is also more costly. Contingent NIPT may provide a cost-effective alternative to universal NIPT screening. Contingent screening used a two-stage process in which risk is assessed by MSS in the first stage and, based on a risk cutoff, high-risk pregnancies are referred for NIPT. The objective of this study was to (1) determine the optimum MSS risk cutoff for contingent NIPT and (2) compare the cost effectiveness of optimized contingent NIPT to universal NIPT and conventional MSS.

**Study Design:**

Decision-analytic model using micro-simulation and probabilistic sensitivity analysis. We evaluated cost effectiveness from three perspectives: societal, governmental, and payer.

**Results:**

From a societal perspective, universal NIPT dominated both contingent NIPT and MSS. From a government and payer perspective, contingent NIPT dominated MSS. Compared to contingent NIPT, adopting a universal NIPT would cost $203,088 for each additional case detected from a government perspective and $263,922 for each additional case detected from a payer perspective.

**Conclusions:**

From a societal perspective, universal NIPT is a cost-effective alternative to MSS and contingent NIPT. When viewed from narrower perspectives, contingent NIPT is less costly than universal NIPT and provides a cost-effective alternative to MSS.

## Introduction

Non-invasive prenatal testing (NIPT) is a relatively new screening method that provides greater prenatal detection of aneuploidies than conventional maternal serum screening (MSS). Clinical trial results show that NIPT is both sensitive and specific. For trisomy 21 (Down syndrome), NIPT has a sensitivity (detection rate) and specificity of approximately 99%.[1] Thus, NIPT provides high accuracy without the risk associated with invasive diagnostic testing (amniocentesis, chorionic villus sampling). NIPT can provide results as early as the 10th week of pregnancy.

Although NIPT is more accurate than MSS, it is also costly. Current list prices range from $500 to $2100 per test.[2,3] Despite the cost, studies have shown that universal NIPT screening (i.e., replacement of MSS by NIPT) is cost effective when viewed from a societal perspective.[4] Although the societal perspective is preferred for theoretical reasons, most decision makers actually use narrower perspectives such as governmental or payer perspective. Universal NIPT is not cost effective when viewed from these narrower perspectives.[4] NIPT-based screening policies might be acceptable if NIPT were used in a select subset of pregnancies rather than applied universally. Indeed, studies have shown that the selective use of NIPT among higher risk women (contingent NIPT) is less costly than universal NIPT.[5,6]

Contingent NIPT policies use a two-stage screening strategy. In the first stage, MSS is used to estimate the probability of an affected pregnancy. A pregnancy is categorized as “high risk” based on a risk threshold. Patients are referred for NIPT only if the probability of an affected pregnancy is greater than the risk threshold. Therefore, NIPT is *contingent* upon the results of the primary screen.

Contingent NIPT strategies are less costly than universal NIPT screening because a relatively small subset of “high-risk” patients are referred for NIPT testing.[5–7] Contingent NIPT screening policies can achieve higher detection rates than MSS by using lower risk cutoffs in the first stage. Lowering the risk cutoff in the first stage increases the number of cases classified as “high risk” so that a greater percentage of cases are referred to NIPT testing. Lowering the risk cutoff increases sensitivity but also increases false positives; however, NIPT is very specific,[1] so most false-positive results obtained in the first stage are identified in the second stage. In addition, positive results from the first stage could be followed by reflexive NIPT. When tested this way, contingent NIPT would spare women the anxiety associated with false-positive results.[7] Because it is applied to a small subset of pregnancies, contingent NIPT has the potential to reduce costs relative to universal NIPT screening with little loss of accuracy. Thus, contingent NIPT may be a cost-effective alternative to universal NIPT and MSS.

The risk cutoff of the primary screen is a key design factor for contingent NIPT policies because it affects both screening performance (sensitivity and specificity) and downstream costs. Thus, it is important to determine the best cutoff point of the primary screen to optimize the overall cost-effectiveness of a contingent screening policy. We refer to such a screening process as an *optimized contingent NIPT*. To our knowledge, the optimal cutoff has not been determined.

Several studies have examined the cost effectiveness of contingent screening policies. There have been two approaches. Some studies used risk cutoffs similar to those used in MSS [8–11] where others compared a wide range of risk cutoffs. [5–7] Neither of these approaches uses an optimized cutoff. Studies to identify the optimal risk cutoff of the primary screen are needed to compare MSS and universal NIPT to the best available contingent NIPT policy.

The optimal cutoff for contingent screening balances screening costs (cases referred to second stage NIPT) and downstream costs (medical costs, productivity, indirect costs). Some downstream costs are only relevant in particular economic perspectives (societal, government, payer). Therefore, the optimal cutoff point of a contingent screening policy depends on the economic perspective. The evaluation of contingent NIPT relative to alternatives (MSS, universal NIPT) should be based on contingent NIPT policies that are optimized relative to a particular economic perspective.

This study compared the cost effectiveness of contingent NIPT to MSS and universal NIPT using three different economic perspectives: societal, governmental, and payer. For each perspective, we determined the optimal cutoff of the primary screen and then compared the performance of the optimized contingent NIPT policy to MSS and universal NIPT.

## Methods

### Design

**Fig 1:**
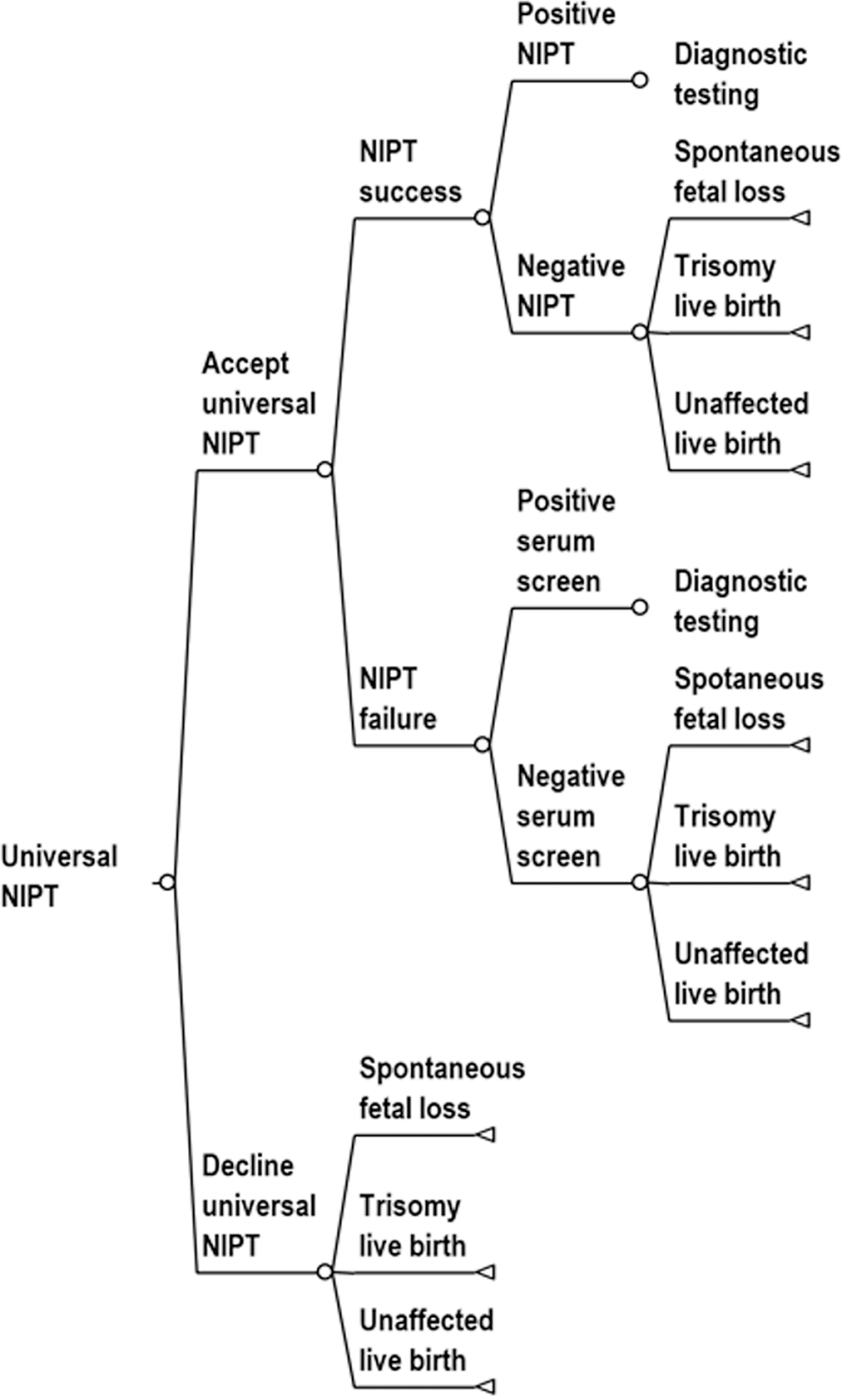
Decision tree diagram for universal NIPT. We assumed that women with failed NIPT would be tested with serum screening and that women with a serum screen risk greater or equal to 1:270 would be offered diagnostic testing. The decision tree is continued in the diagnostic testing tree (Fig 4).

**Fig 2:**
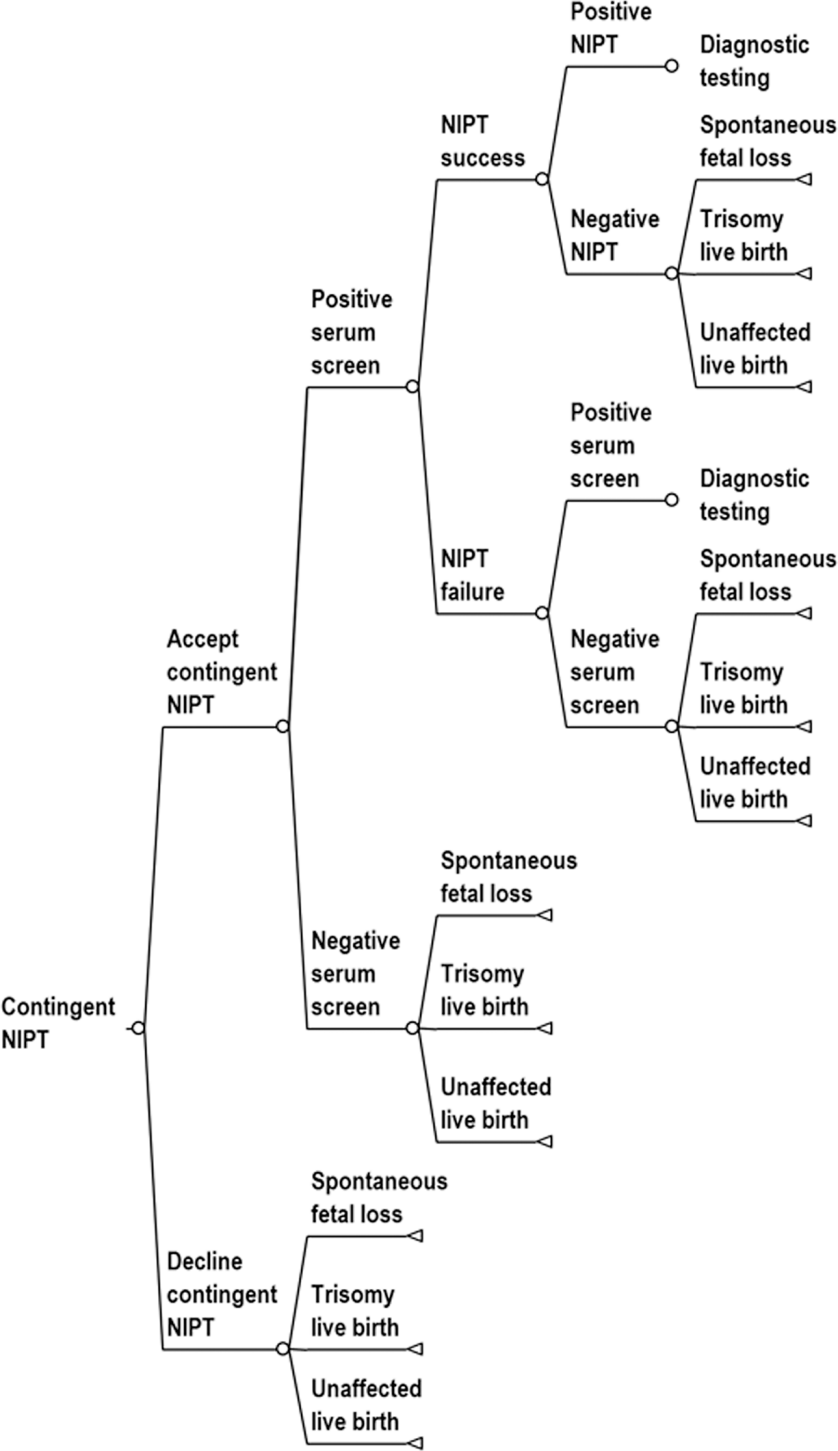
Decision tree diagram for contingent NIPT. We assumed that women with failed NIPT whose risk was higher than or equal to 1:270 on the initial serum screen would be offered diagnostic testing. The decision tree is continued in the diagnostic testing tree (Fig 4).

**Fig 3:**
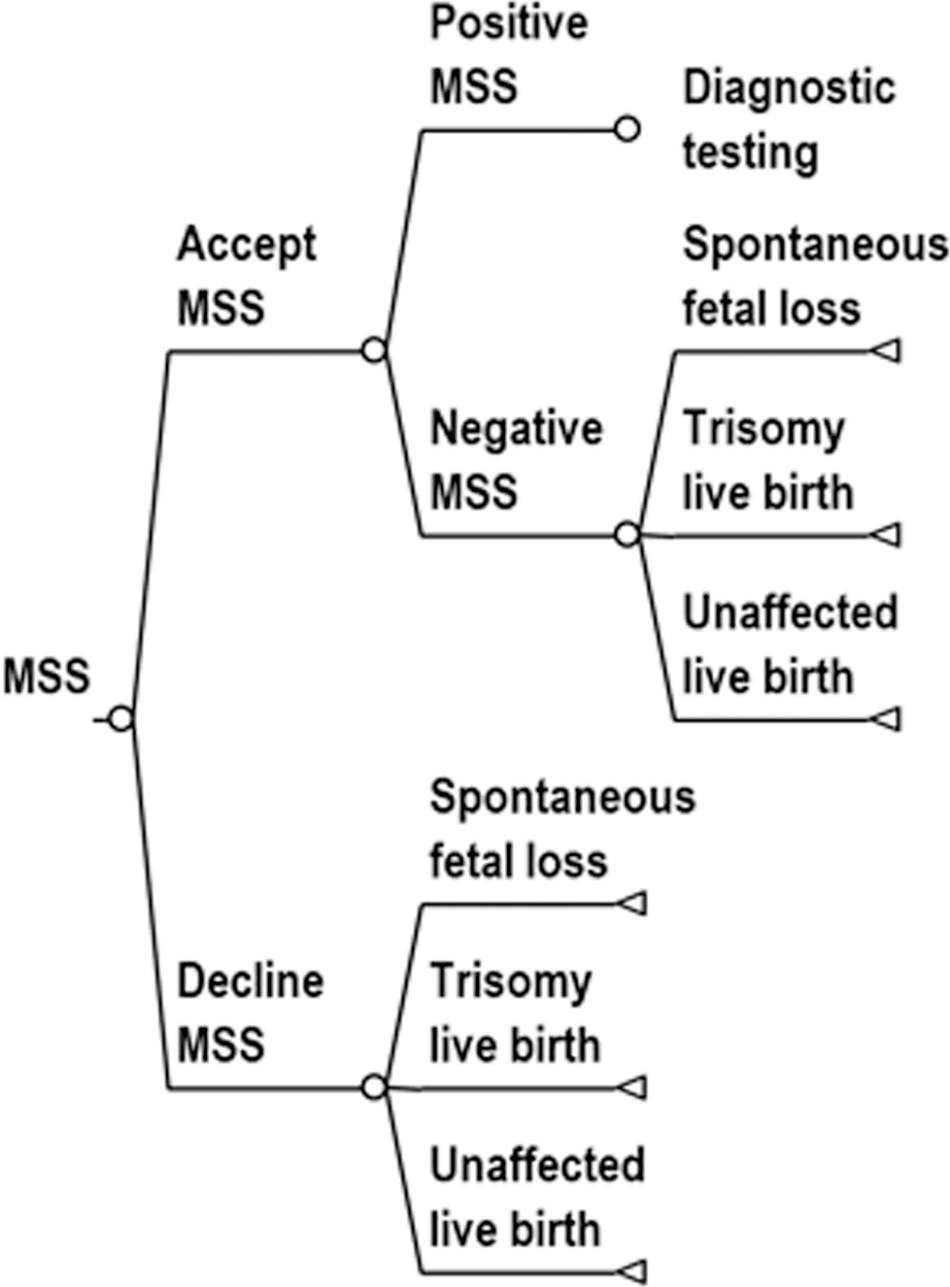
Decision tree diagram for MSS. The decision tree is continued in the diagnostic testing tree (Fig 4).

**Fig 4:**
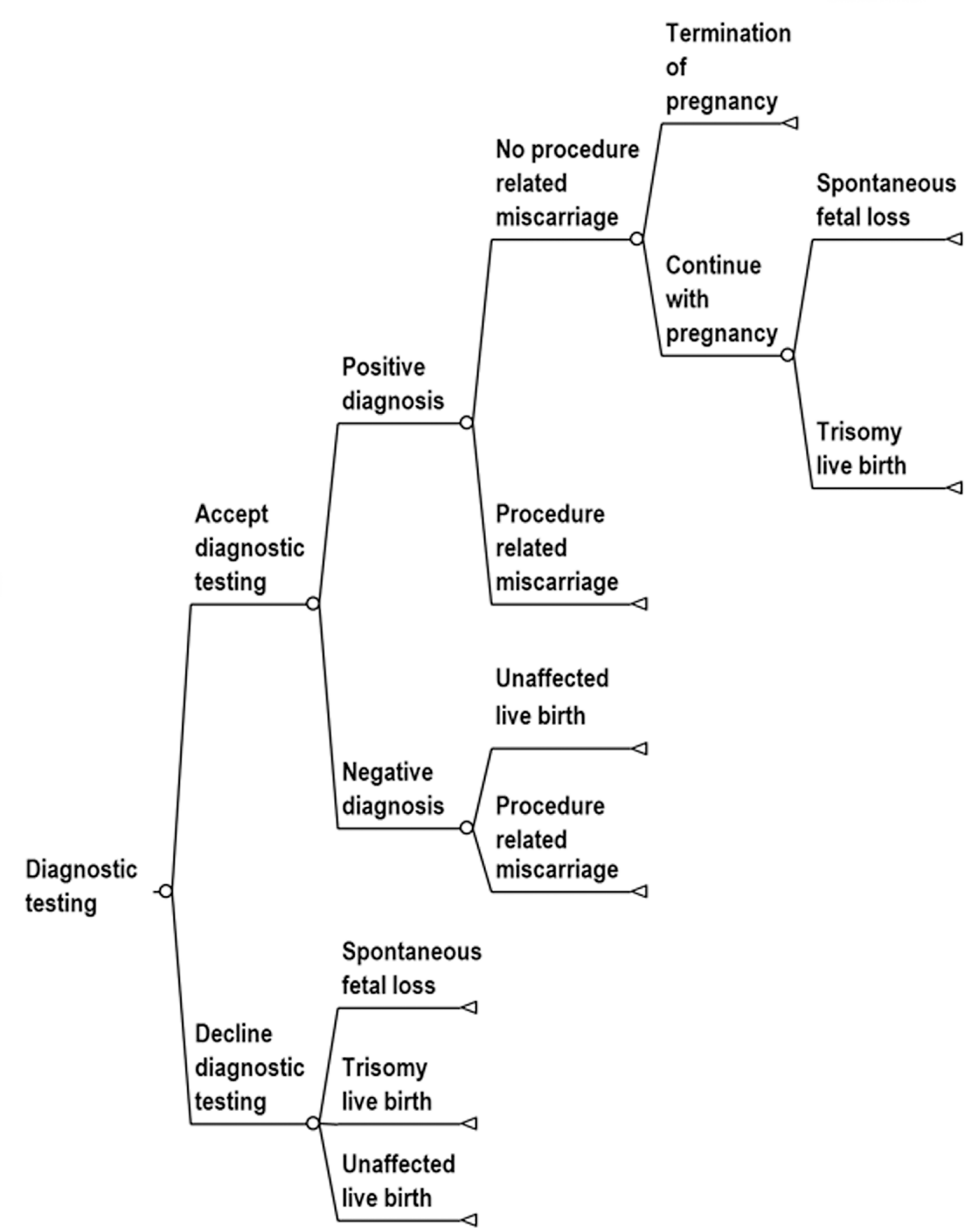
Decision tree diagram for diagnostic testing.

We studied the cost effectiveness of routine use of contingent NIPT policies relative to conventional MSS and universal NIPT using a simulated population designed to represent the general population of women in the United States. We assumed that MSS and contingent NIPT used the combined serum test. We used a decision-analytic model because prenatal testing can be represented by a relatively simple sequence of decisions: Decision tree diagrams for screening protocols are provided in Figs 1–4. We based the cost effectiveness analysis upon a hypothetical cohort, and it is therefore exempt from institutional review board approval.

### Perspective and time horizon

Our analysis included a societal perspective as recommended by many cost-effectiveness guidelines.[12,13] Although a societal perspective is recommended, decisions are often based on narrower perspectives and, for that reason, we also included government and payer perspectives. The societal perspective included immediate costs of screening and the direct and indirect lifetime costs. The government perspective included the immediate screening costs and direct lifetime medical and education costs. The payer perspective included only the immediate costs associated with screening.

### Standard of care and comparator

We used MSS as a standard of care using the combined test (using pregnancy-associated plasma protein-A, free beta human chorionic gonadotropin and nuchal translucency ultrasound measurements). For this, we used a 2^nd^ trimester risk cutoff of 1:270 for trisomy 21 and 1:100 for trisomy 18 and 13. We compared MSS to three alternatives: (1) universal NIPT, (2) contingent NIPT, and (3) no screening.

### Optimization of risk cutoff

We determined the optimal cutoff by minimizing the expected total cost by simultaneously varying the decision thresholds for all three tests subject to the constraint that the detection rate had to be at least equivalent to conventional material screening. Because costs varied by perspective, optimal risk cutoffs were determined for each perspective. The optimization can be expressed as follows:

$${TC}_{i}^{*}=TC(L_{13,i}^{*}\;L_{18,i}^{*},L_{13,i}^{*},C_{i},p)=\underset{L_{13,i},L_{18,i},L_{21,i}}{\min}TC(L_{13,i},L_{18,i},L_{21,i},C_{i},p)$$ 1A

$$s.t.\;{DR}_{cont}(L_{13,i},L_{18,i},L_{21,i})>{DR}_{MSS}$$ 1B

Where *TC*_*i*_ = expected total cost under economic perspective *i*, *L*_*j*,*i*_ is the decision threshold for marker *j* under perspective *i*, *C*_*i*_ is the cost of an affected pregnancy under perspective *i*, *DR*_*cont*_ is the first stage detection rate for contingent screening, *DR*_*MSS*_ is the detection rate of conventional MSS, and *p* is the vector of additional parameters that are invariant to the economic perspective (e.g., uptake rates, test accuracy, etc). The asterisks designate optimal values. Because conventional MSS is the current standard of care, we reasoned that a screening policy with lower accuracy would not be acceptable. We therefore constrained the optimization of contingent NIPT to meet or exceed the detection rate of current practice. We performed the optimization using a grid search. We generated a large grid of cost and threshold combinations. The optimization was performed in stages. Once we identified the neighborhood of the optimum, we created successively finer grids to identify the optimum risk thresholds. Grids were generating using @Risk (Palisade Corp, Ithaca, NY).

All results for contingent screens were based on optimized cutoffs. For simplicity, we will refer to an optimized contingent policy as a contingent NIPT policy.

### Screening performance

We used population parameters from the Serum Urine and Ultrasound Screening Study (SURUSS) to estimate screening performance of maternal serum screening for trisomy 21[14,15] and used populations parameters from another study to estimate serum screening performance for trisomy 18 and 13.[16] Age-specific detection and false positive rates of serum screening was estimated by a Monte Carlo simulation performed using Stata 12.1.[17] Multiples of median, standard deviations, correlation coefficients, and truncation limits provided by SURUSS were used to create multivariate Gaussian distribution to simulate 1,000,000 marker sets of affected and unaffected pregnancies. Post-test risks were derived from the likelihood ratios and prior risk.[18–20] For conventional maternal serum screening, a second trimester risk cutoff of 1:270 was used for trisomy 21 and a 1:100 risk cutoff was used for trisomy 18 and 13. Screen results were classified as “positive” if the post-test risk was equal to or greater than the specified risk cutoff and negative otherwise. Based on those results, age-specific detection and false-positive rates for the risk cutoffs were calculated. Detection rates of 99%, 96.8% and 92.1% were assumed for trisomy 21, 18, and 13 based on a meta-analysis of NIPT performance.[1] An overall false positive rate of 0.41% for unaffected pregnancies was assumed based upon the individual reported false positive rates for trisomy 21, 18, and 13 reported in the same study. We assumed a 2.8% failure rate for NIPT due to low fetal fraction or assay failure based on a weighted average of studies using maternal blood drawn between 10–13 weeks of pregnancy.[21–24]

### Cost-effectiveness simulation

Our cost-effectiveness simulation was based on commonly used modeling practices, which incorporated a probabilistic sensitivity analysis and micro-simulation.[12] The probabilistic sensitivity analysis was conducted by repeating the micro-simulation 1,000 times. During each iteration of the probabilistic sensitivity analysis, the model costs and probabilities were randomly drawn and a micro-simulation was completed using the drawn values. Following standard practice, the costs were drawn from gamma distributions while the probabilities were drawn from beta distributions.[25] The definitions of beta and gamma distributions are provided in Table 1. The micro-simulations were conducted by simulating 1,000,000 pregnant women at 12 weeks of pregnancy. For each simulated woman, a maternal age was assigned based upon the maternal age distribution reported in the 2012 National Vital Statistics birth data.[26] The individual risk of an affected pregnancy, detection rate, and false-positive rate was determined by maternal age.

**Table 1:** Model probabilities and costs.

Probabilities	Mean	95th% CI	Parameters of beta distribution	
			alpha	beta
MSS uptake, *U*_*MSS*_	69%	64%-74%	226.104	101.583
Increase in contingent NIPT uptake over MSS, *ΔU*_*cNIPT*_	8.2%	4.6%-12.6%	14.606	163.516
Increase in universal NIPT uptake over MSS, *ΔU*_*uNIPT*_	13.5%	7.6%-20.8%	13.705	87.814
Diagnostic testing uptake	66%	61%-71%	226.923	116.899
Procedure-related fetal loss	0.22%	0%-1.16%	0.447	202.595
Termination rate of trisomy 21	80%	74%-86%	135.790	33.948
Termination rate of trisomy 18	80%	73%-87%	99.552	24.888
Termination rate of trisomy 13	92%	85%-97%	71.336	6.203
NIPT detection rate of trisomy 21	99%	98.3%-99.5%	1,044.886	10.554
NIPT detection rate of trisomy 18	96.8%	95%-98.2%	448.991	14.843
NIPT detection rate of trisomy 13	92.1%	86.9%-96.1%	120.738	10.356
NIPT false positive rate	0.41%	0.29%-0.55%	36.775	8,944.280
NIPT failure rate due to low fetal fraction	2.8%	1.2%-5.1%	7.291	253.092
Costs	Mean	95th% CI	Parameters of gamma distribution	
			alpha	beta
Combined screen	$166	$95-$257	16	10.375
Cost of NIPT	$400	$229-$619	16	25
Cost of CVS	$1,010	$577-$1,562	16	63.125
Cost of genetic counseling	$160	$91-$247	16	10
Termination of pregnancy	$581	$332-$898	16	36.313
Direct lifetime costs of trisomy 21	$427,577	$244,397-$661,147	16	26,723.563
Indirect lifetime costs of trisomy 21	$1,069,195	$611,137-$1,653,257	16	66,824.688
Direct lifetime costs of trisomies 13 and 18	$37,971	$21,704-$58,713	16	2,373.188
Indirect lifetime costs of trisomies 13 and 18	$1,363,877	$779,574-$2,108,913,	16	85,242.313

A standard deviation of 25% of the mean was assumed for the percentage increases for contingent NIPT and universal NIPT uptake. As standard practice, normal distributions of probabilities were approximated with beta distributions. Normal distributions of costs were approximated with gamma distributions

The effectiveness measure was the number of affected pregnancies detected. Simulation analyses were performed with TreeAge Pro 2012 software.[27]

### Costs

We included the costs of screening, diagnosis, and termination of pregnancy as well as the lifetime costs associated with trisomy 21, 18, and 13. Lifetime costs represent the average difference in direct medical and educational costs between trisomy and an average individual in addition to the indirect costs of lost productivity due to morbidity and mortality associated with this syndrome.

We derived lifetime and termination costs from the literature.[28,29] Lifetime costs for trisomy 21 by Waitzman et al. were updated by using more recent survival data.[30,31] We assumed that the annual costs of trisomies 13 and 18 were the same as the average annual costs of trisomy 21. We were not able to find survival data for trisomy 13 past the first year. We therefore assumed that the survival for trisomy 13 was the same as the survival for trisomy 18 past the first year.[32,33] We inflated lifetime costs and the cost of termination to reflect 2013 US dollars. We adjusted the medical portion of lifetime costs using the health care component of the personal consumption expenditure index.[34] We adjusted the non-medical direct and indirect portions of lifetime costs using the employment cost index for civilian workers.[35] As recommended, future costs incurred beyond one year were discounted to present value using an annual rate of 3%.[12,13] Additional explanation of the lifetime cost estimates is provided in S1 Text.

The costs of serum screenings, diagnostic testing, and genetic counseling were derived from the 2013 Medicare Physician Fee Schedule (MPFS),[36] which is often used to approximate the resource value of medical care.[13] These costs included procedure costs as well as genetic counseling.

The cost of NIPT testing is uncertain. The list price can serve as an indicator of cost, but list prices show wide variation. Because NIPT providers utilize similar techniques, we would expect the underlying costs to be similar for all test providers. Therefore, the variation in price is more likely an indication of variation in profit margins rather than variation in costs. For genetic testing, these margins can be as high as 90%.[37] For that reason, we based the resource cost of NIPT on the lowest priced test (NIFTY offered by Beijing Genomics Institute in the UK for approximately $500). We assumed a conservative profit margin of 20% for a unit cost of $400.[2] This cost is consistent with cost estimates published in previous cost-effectiveness studies.[4,38]

We assumed costs had a standard deviation that was 25 percent of the mean. Gamma cost distributions were estimated using the mean and standard deviation values.[25] The costs are summarized in Table 1.

### Baseline model

Baseline model inputs are shown in Table 1. The baseline uptake rate for conventional screening was assumed to be 69%.[39] For contingent and universal NIPT, we assumed screening uptake increased by 8.2% and 13.5% relative to MSS, based upon a recent study of potential uptake of NIPT.[40] Therefore, we modeled contingent and universal NIPT uptake as follows:

$$U_{cNIPT}=U_{cMSS}\;*\;(1+{\Delta U}_{cNIPT})$$ 2A

$$U_{uNIPT}=U_{cMSS}\;*\;(1+{\Delta U}_{uNIPT})$$ 2B

Where *U*_*cMSS*_ is the baseline uptake based on conventional MSS, *U*_*cNIPT*_ and *U*_*uNIPT*_ are the uptake for contingent NIPT and universal NIPT. *ΔU*_*cNIPT*_ and *ΔU*_*uNIPT*_ are the fractional change in uptake relative to *U*_*cMSS*_. We assume that *ΔU*_*cNIPT*_ and *ΔU*_*uNIPT*_ are 0.082 and 0.135. This resulted in a baseline uptake of approximately 75% for contingent NIPT and 78% for universal NIPT.

For contingent screening, we assumed that all primary screens exceeding the risk threshold (i.e., “positive” on the primary screen) were followed by reflexive NIPT. A contingent NIPT screen was classified as positive only if the primary and secondary screens were both positive. We assumed that all positive screen results (MSS, contingent NIPT, universal NIPT) were followed by diagnostic testing at an acceptance rate of 66%.[41–46] We assumed that the termination rates for pregnancies diagnosed as positive were 80% for trisomy 21,[47–59] 80% for trisomy 18,[48,60,61] and a rate of 92% for trisomy 13.[52,60] We assumed spontaneous fetal loss rates 43%,[62] 72%,[63] and 49%[63] for trisomy 21, 18, and 13 respectively. Procedure-related fetal loss from chorionic villus sampling (CVS) was assumed to be 0.22% based on a recently published meta-analysis.[64]

We assumed that the technical failure rate of NIPT was 2.8%.[21–24] To our knowledge, there is no standard protocol for NIPT failure. For simplicity, we assumed NIPT would not be repeated in the event of an NIPT test failure. In this event, we assumed that women who elected contingent NIPT would be referred to invasive diagnostic testing based on MSS results, For those who elected universal NIPT, failures would be referred for MSS, and those who were classified as high risk (i.e., those with a trisomy 21 risk equal to or greater than 1:270 or a trisomy 13 or 18 risk equal to or greater than 1:100) would be referred for invasive diagnostic testing.

### Sensitivity analysis

Sensitivity analysis was performed using one-way and probabilistic sensitivity analysis. One-way sensitivity analyses were conducted to determine the individual impact of each input parameter value on cost-effectiveness ratios. Probabilistic sensitivity analysis was conducted to determine the overall uncertainty in the cost effectiveness due to the combined impact of uncertainty in the underlying model inputs. The parameters of the distributions are reported in Table 1. The objectives and, therefore, the parameters, of a one-way sensitivity analysis differ from the objectives of a probabilistic sensitivity analysis. For example, a one-way analysis may be designed to determine the point at which the best strategy changes (which are not necessarily plausible), whereas a probabilistic sensitivity should investigate the sensitivity over plausible ranges of the input variables.

#### Terminology

A screening policy is said to be *strictly dominated* by another policy if it is both more costly and more expensive.[12] A policy is *dominated by extension* if a combination of alternatives is less costly.[12] For example, policy A is dominated by extension by policies B and C if, on average, a combination of policies B and C (X% policy B, 1-X% policy C) is less expensive and more effective than policy A.

Because there is no agreed-upon willingness-to-pay threshold for trisomy screening, we deemed NIPT strategies as cost effective if they dominated MSS (the current standard of care) strictly or by extension.

## Results

The prevalence at 12 weeks was approximately 1 in 301 for trisomy 21, 1 in 1,170 for trisomy 18, and 1 in 3,627 for trisomy 13. In the absence of screening, this resulted in a lower birth prevalence of 1 in 528, 1 in 4,174, and 1 in 7,084 live births for trisomy 21, 18, and 13 respectively due to spontaneous fetal loss. These rates are consistent with reported birth prevalence for these trisomies.[19] The optimal risk cutoffs, percentage of women screened receiving NIPT, detection rates, false positive rates, and number of failed NIPTs are provided in Tables 2 and 3. Total costs, cases detected, and incremental cost effectiveness ratios (ICERs) are provided in Table 4.

**Table 2:** Optimal risk cutoffs and the number of women receiving NIPT for contingent NIPT policies.

	Optimal Risk Cutoff			
Perspective	Trisomy 21	Trisomy 18	Trisomy 13	NIPT referral rate
Societal	1:1515	1:1905	1:860	24.0%
Government	1:420	1:145	1:175	8.7%
Payer	1:315	1:115	1:175	7.0%

**Table 3:** Detection rates, false positive rates, optimal risk cutoffs, and NIPT failure rates.

	Detection rates			False positive rates	NIPT failure rates
	Trisomy 21	Trisomy 18	Trisomy 13		
Universal NIPT	99%	96.8%	92.1%	0.4%	2.8%
MSS	84.8%	75.8%	62.8%	5.6%	0%
Contingent NIPT					
Societal perspective	93.6%	92.7%	77.7%	0.094%	0.66%
Government perspective	87%	82.1%	63.3%	0.033%	0.24%
Payer perspective	85.1%	75.6%	63.3%	0.026%	0.19%

**Table 4:** Total cost, cases detected, incremental costs, incremental cases detected and incremental cost effectiveness ratio (ICER).

	Total cost	Cases detected	Incremental costs	Incremental cases detected	ICER
Societal perspective					
No screening	$3,347,297,152	0			Strictly dominated
MSS	$2,475,580,143	2,516			Strictly dominated
Contingent NIPT	$2,315,959,639	3,077			Strictly dominated
Universal NIPT	$2,305,749,493	3,409			
Government perspective					
No screening	$822,000,565	0			Strictly dominated
MSS	$711,465,188	2,516			Strictly dominated
Contingent NIPT	$693,996,197	2,817			
Universal NIPT	$814,224,159	3,409	$120,277,962	592	$203,088
Payer perspective					
No screening	$0	0			
MSS	$142,723,273	2,516			Dominated by extension
Contingent NIPT	$148,208,927	2,729	$148,208,927	213	$25,754
Universal NIPT	$327,675,783	3,409	$179,466,856	680	$263,922

### Societal perspective

Both direct and indirect lifetime costs were included in the analysis from a societal perspective. The optimized contingent NIPT screening policy had detection rates of 93.6%, 92.7%, and 77.7%, for trisomy 21, 18, and 13 respectively. In contrast, the detection rates were 84.4%, 75.8%, and 62.8% for conventional MSS and 98.7%, 96.4%, and 91.5% for universal NIPT. Of women screened with contingent NIPT, approximately 24% were classified as “high risk” in the primary screen and referred for NIPT. Contingent NIPT had a false positive rate of 0.09%, conventional MSS had a false positive rate of 5.6%, and universal NIPT had a false positive rate of 0.9%. Contingent NIPT also had fewer test failures than universal NIPT. Only 0.66% of those screened with contingent NIPT had a technical failure whereas 2.8% of women screened with universal NIPT failed to obtain a result.

No screening, MSS, and contingent NIPT were all dominated by universal NIPT. Out of 1,000,000 pregnancies, replacing MSS with universal NIPT would result in an increase of 893 detections and a cost savings of approximately $170 million (Table 4).

**Fig 5:**
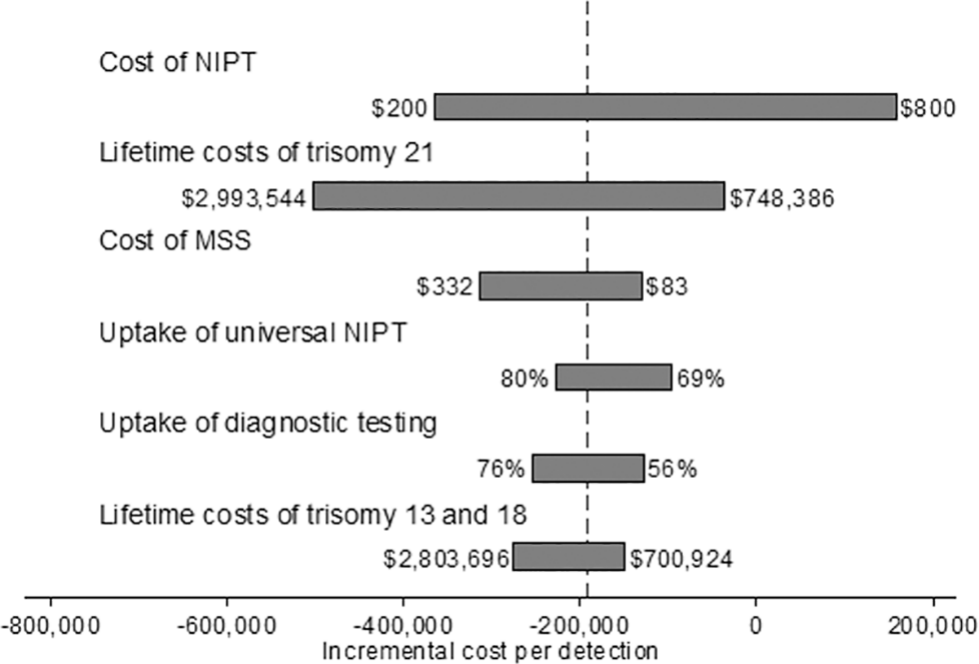
One-way sensitivity analysis of universal NIPT vs MSS from a societal perspective. Below are the one-way sensitivity analysis results of the ICER between universal NIPT and MSS. Universal NIPT is less costly than MSS as long as the cost of NIPT remains below $619.

**Fig 6:**
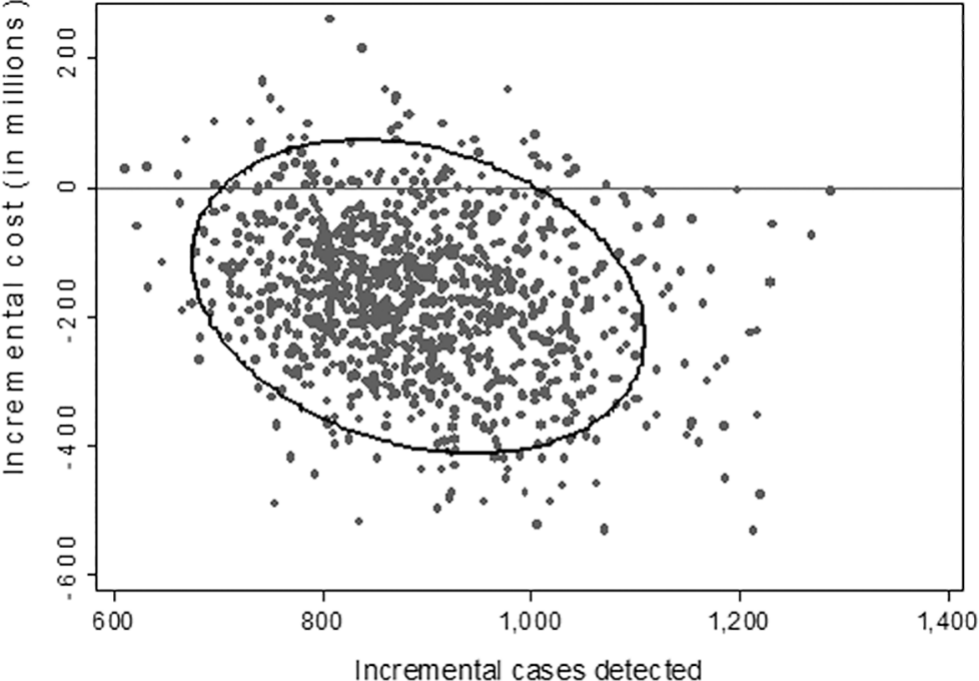
Scatter plot of probabilistic sensitivity analysis, universal NIPT vs MSS. The figure below plots the incremental cost and effectiveness results from 1,000 simulations. Compared to MSS, there is a 100% probability that universal NIPT is more effective and 91.8% probability that universal NIPT is less costly.

We conducted one-way sensitivity analysis of the ICERs (Fig 5). Universal NIPT remained less costly than conventional MSS so long as the cost of NIPT was below $619. In the probabilistic sensitivity analysis, universal NIPT was more effective 100% of the time and less costly 91.1% of the time compared to MSS (Fig 6).

### Government perspective

Indirect costs were not included in the government perspective. When indirect lifetime costs were excluded from the analysis, the contingent NIPT policy had detection rates of 87%, 82.1%, and 77.7%, for trisomy 21, 18, and 13, a false positive rate of 0.033%, and a failure rate of 0.24%. Approximately 8.7% of women screened by contingent NIPT received NIPT after the initial screen.

**Fig 7:**
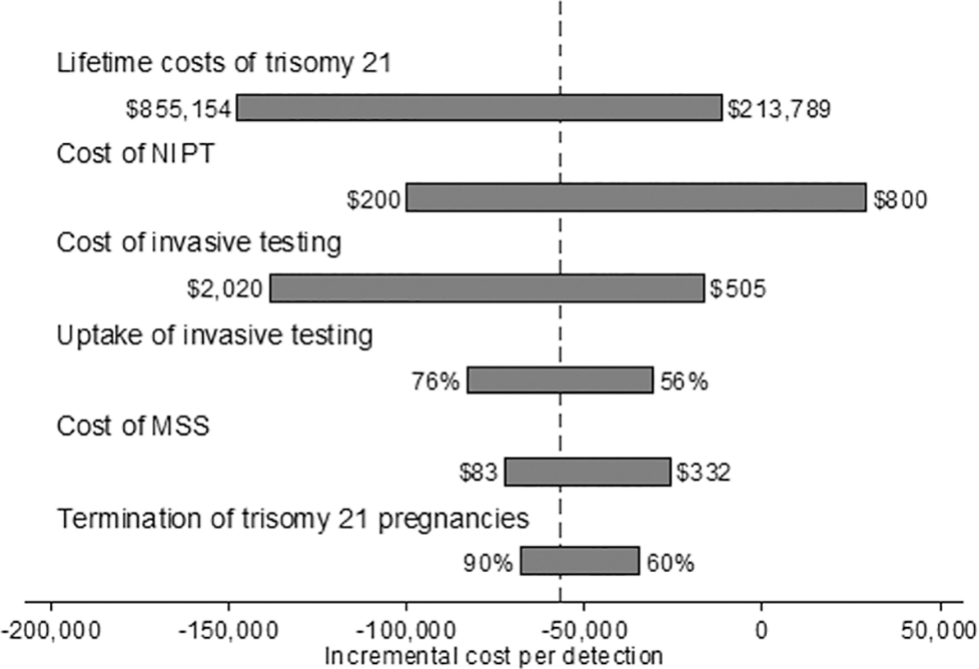
One-way sensitivity analysis of contingent NIPT vs MSS from a government perspective. Below are the one-way sensitivity analysis results of ICER between contingent NIPT and MSS. Contingent NIPT is less costly than MSS as long as the cost of NIPT remains below $663.

**Fig 8:**
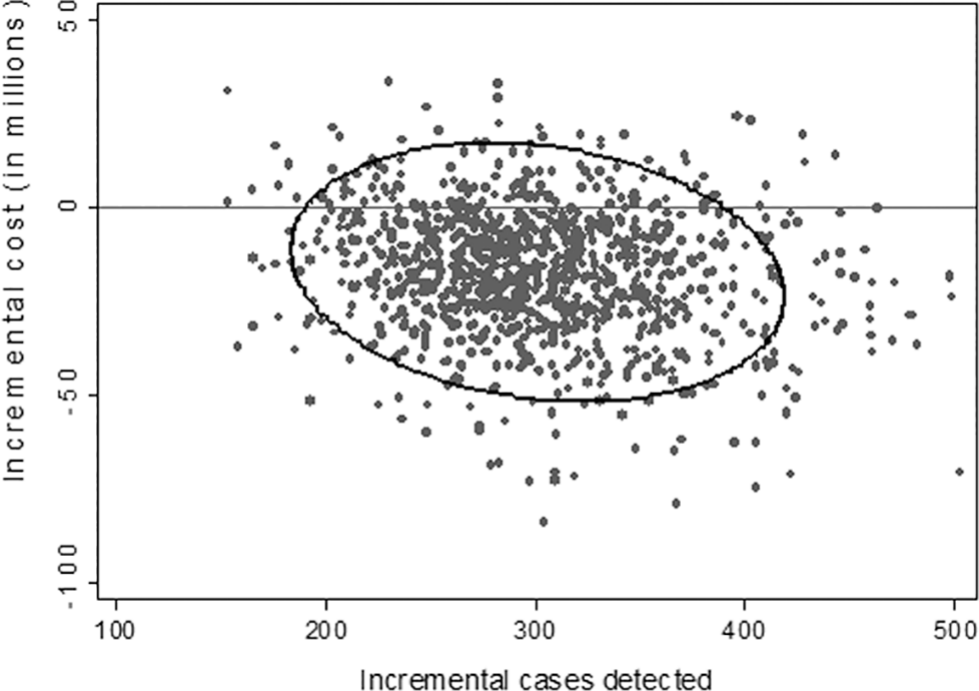
Scatter plot of probabilistic sensitivity analysis of contingent NIPT vs. MSS from a government perspective. The figure below plots the incremental cost and effectiveness results from 1,000 simulations. Compared to MSS, there is a 100% probability that contingent NIPT is more effective and a 87% probability that contingent NIPT is less costly.

Contingent NIPT dominated MSS when evaluated from the government perspective. Out of 1,000,000 pregnancies, replacing combined MSS with contingent NIPT would result in an increase of 301 detections and a cost savings of approximately $17.5 million (Table 4). Universal NIPT was more effective but also more costly than contingent NIPT. Universal NIPT would increase the number of cases detected by contingent NIPT by 592 and increase costs by $120 million, for an ICER of $203,088 per additional case detected. The one-way analysis shows that contingent NIPT screening dominated MSS unless the cost of NIPT exceeded $663 (Fig 7). In the probabilistic sensitivity analysis, contingent screening was more effective 100% of the time and less costly 87% of the time compared to MSS (Fig 8).

### Payer perspective

When all lifetime costs were excluded from the analysis, the optimal risk cutoffs resulted in detection rates of 85.1%, 75.8%, and 63.3%, for trisomy 21, 18, and 13, respectively, a false positive rate of 0.026%, and a failure rate of 0.19%. Approximately 7% of women screened with the contingent NIPT received NIPT after the initial screen.

No screening is the least costly strategy, followed by MSS, contingent NIPT, and universal NIPT. Although contingent NIPT is more effective and more costly than MSS in terms of cost per additional detection, contingent NIPT is a more efficient strategy (Table 4). Compared to no screening, MSS would cost $56,726 per case detected; however, contingent NIPT would cost $54,309 for each detection. Therefore, contingent NIPT dominates MSS by extension.

**Fig 9:**
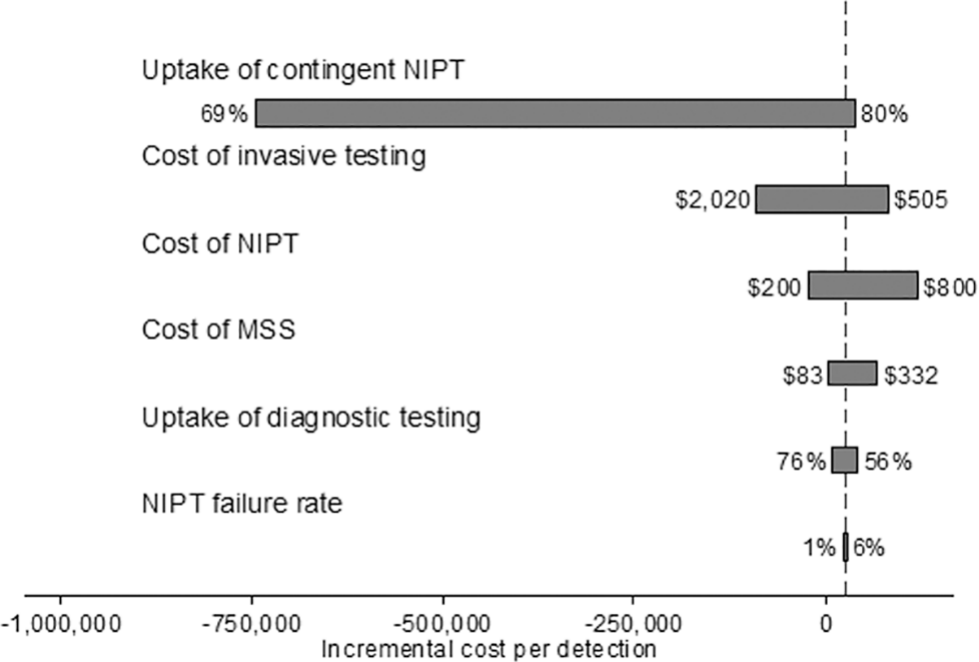
One-way sensitivity analysis of contingent NIPT vs MSS from a payer perspective. Below are the one-way sensitivity analysis results of ICER between contingent NIPT and MSS. Contingent NIPT is more costly than MSS as long as the cost of NIPT is above $293, contingent NIPT uptake is above 72%, and the cost of invasive screening is below $1,235.

**Fig 10:**
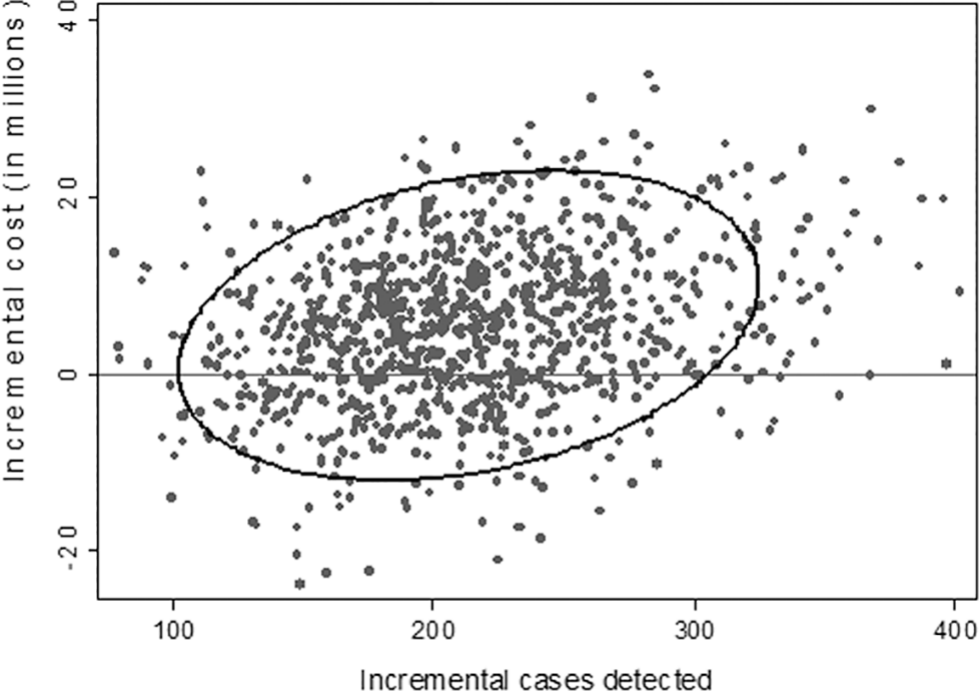
Scatter plot of probabilistic sensitivity analysis of contingent NIPT vs MSS from payer perspective. The figure below plots the incremental cost and effectiveness results from 1,000 simulations. Compared to MSS, there is a 100% probability that contingent NIPT is more effective but a 73.2% probability that contingent NIPT is more costly.

Compared to contingent NIPT, universal NIPT would increase the number of cases detected by 680 and increase costs by $179 million, for an ICER of $263,922 per additional case detected. The one-way analysis shows contingent NIPT was less costly than MSS when (1) the cost of NIPT was below $293, (2) contingent NIPT uptake was below 72%, and (3) the cost of invasive screening was above $1,235 (Fig 9). In the probabilistic sensitivity analysis, contingent screening was more effective 100% of the time and more costly 73.2% of the time compared to MSS (Fig 10).

## Discussion

We compared the cost effectiveness of four screening policies to identify fetal trisomies: (1) no screening, (2) conventional MSS, (3) universal NIPT screening, and (4) optimized contingent NIPT. We conducted our analysis from three different perspectives: societal, government, and payer.

We optimized the risk cutoff used to classify “high-risk” pregnancies in the primary stage of the contingent NIPT screen. We found that the optimal risk cutoff depended on the cost perspective. The optimal risk cutoff of the primary stage decreased when more downstream costs were included in the analysis. Lower risk cutoffs in the first stage increased the referral rate to the second stage (NIPT) which, in turn, increased the detection rate. The risk cutoffs were lowest when optimized for the societal perspective and highest when optimized for the payer perspective.

We found that the best screening policy depended on the economic perspective. When analyzed from a societal perspective, universal NIPT was both more effective and less costly than contingent NIPT. Universal NIPT was also less costly than MSS. Replacing MSS with universal NIPT would result in a 35.5% increase in total cases detected and 6.9% reduction in total costs.

From a government perspective, contingent NIPT dominated MSS and was the least costly strategy. Under this perspective, the optimized risk cutoffs would result in a 12% increase in the number of total cases detected and a 2.5% reduction in costs. In contrast to a societal perspective, the government perspective excluded the indirect costs from the analysis. When indirect costs are no longer included, universal NIPT is not a cost-effective alternative to MSS unless there is a substantial willingness to pay for the test. For universal NIPT to be a cost-effective replacement for MSS, there must be a willingness to pay of $203,088 per detection.

From a payer perspective, MSS is the least costly screening strategy. Although MSS was less costly, contingent NIPT dominated MSS by extension. The ICER between no screening and contingent NIPT was $54,309 per case detected; however, a comparison of no screening and MSS produces an ICER of $56,726 per case detected. Given that MSS is currently the standard of care, we can reasonably assume that there is at least a willingness to pay of $56,726 per case detected, making a contingent NIPT a cost-effective alternative to MSS from a payer perspective.

Contingent and universal NIPT produced fewer false positive results than MSS. MSS had a false positive rate of 5.6%, universal NIPT had a false positive rate of 0.4%, and all contingent NIPT polices had false positive rates below 0.1%. Thus, contingent NIPT can obtain higher detection rates than MSS with a relatively small increase (0.3%) in the overall false positive rate.

The first stage of contingent NIPT produces a higher level of false positive results than conventional MSS. Unlike conventional MSS, most false positives from the first stage of contingent screening are identified in the second stage so that the overall false positive rate is lower than MSS and only slightly lower than universal NIPT. The high false positive rate of first stage could be a problem if women were provided these results; however, we believe that contingent NIPT should be implemented as a testing system that produces a single test result. Positive results from the first stage would be reflexed to NIPT. Women would only be given the final result and would not be informed of the preliminary false positive result. This approach would spare women the anxiety associated with false positive results.

Previous studies have examined the cost effectiveness of changing the risk cutoff in the primary screen; however, none of these studies included the downstream costs of trisomy births that result from false negative results.[5–7] By including both the immediate costs of screening and the downstream costs of false negative results, we were able to optimize the decision limit. Therefore, our findings provide a new approach to setting the risk thresholds for contingent NIPT.

Our results are consistent with the results of three previous studies that found contingent NIPT to be a cost-effective alternative to conventional MSS.[5,8,9] However, our analysis used multiple perspectives and found risk cutoffs for each perspective that minimized the overall costs of contingent NIPT.

We are unaware of a standard protocol for failed NIPT. We assumed that pregnancies would be classified by conventional MSS in the event of NIPT failure. For contingent NIPT, we assumed that women with a risk greater than or equal to 1:270 on the initial serum screen would be offered invasive testing. Similarly, for universal NIPT we assumed that cases with NIPT failure would be tested with MSS and those with risks greater or equal to 1:270 would be offered invasive testing. When NIPT fails, approximately 56%-83% are successful after another blood draw.[24,65–67] Because some cases are probably redrawn, our analysis probably underestimated the overall detection rate of contingent and universal NIPT. We conducted sensitivity analyses to examine the impact of our assumptions regarding NIPT failure. We found that our results were insensitive to the assumptions. Contingent NIPT was particularly insensitive to this change because only a subset of women initially screened received follow-up NIPT.

The unit cost of NIPT is uncertain. Prices for NIPT testing range from $500 to $2100; however, prices do not necessarily reflect costs. The relevant cost in a cost-effectiveness analysis is the *resource* cost, or the cost to perform the test. Although prices cannot be used directly, we used prices to estimate underlying costs. We reasoned that the producer with the lowest price is most likely making a profit and the variability in price most likely represents variation in profit margins rather than variation in costs. Thus, the lowest price most likely represents an upper bound on the cost. Further, if the variation in prices reflects variation in costs, the lowest cost producer will eventually dominate and is the most relevant cost for the analysis. For that reason, we used the lowest list price ($500) to estimate the cost of NIPT. We assumed a 20% profit margin and used a unit cost of $400 per test. This cost estimate is consistent with previous published results.[4,38] Our sensitivity analysis demonstrated that even at a cost of $800, contingent NIPT was less costly and more effective than conventional screening.

Our analysis assumed that CVS, rather than amniocentesis was used for diagnostic testing. Our model simulates screening at the 12^th^ week. Amniocentesis is usually not performed until the 15^th^ week. We assumed that most women would prefer rapid resolution of a positive screen result and, for that reason, would prefer CVS in the 12^th^ week over amniocentesis at week 15. However, our results are insensitive to this assumption. The number of trisomy cases detected would remain the same while the total costs would be slightly lower due to the small difference in cost between CVS and amniocentesis. Therefore, our conclusions would remain unchanged even if we assumed that diagnostic testing were conducted with amniocentesis.

We conducted extensive sensitivity analysis and probabilistic sensitivity analysis. Our results were most sensitive to changes in the lifetime costs, the increase in uptake of NIPT policies relative to MSS uptake (i.e., *ΔU*_*cNIPT*_ and *ΔU*_*uNIPT*_), uptake of MSS (*U*_*cMSS*_), and the cost of NIPT. Because of the uncertainty regarding the lifetime cost estimates of trisomies 13 and 18, we varied these costs over a wide range in the one-way sensitivity analysis. We found that our results were insensitive to assumptions concerning the lifetime costs associated with trisomies 13 and 18.

Our study had the following limitations regarding the reliability of the lifetime cost estimates. First, the data used to estimate lifetime costs in our analysis is roughly two decades old.[29] Although we adjusted the initial estimates by accounting for inflation and changes in life expectancy, these adjustments do not take into consideration changes in treatment. For example, advances in care may increase the intensity of treatment, therefore increasing lifetime medical expenses.[68] Second, in the absence of lifetime cost estimates for trisomies 13 and 18, we made several simplifying assumptions in order to estimate these costs. Cost data on trisomies 13 and 18 are difficult to obtain. Therefore, we used the annual costs of Down syndrome provided by Waitzman et al. Although trisomies 13 and 18 have similar one-year survival rates,*[32,69–71]* we were unable to find survival estimates beyond one year for trisomy 13; therefore, we assumed that survival rates for trisomy 13 were the same as for trisomy 18 beyond year one.

Because of the uncertainty of the lifetime estimates, we conducted extensive sensitivity analysis on these costs (see Figs 5, 7, and 9). Our conclusions held even when lifetime costs were reduced by 50%, with the following exception. From a societal perspective, universal NIPT no longer dominated contingent NIPT once the lifetime costs of trisomy 21 were below $1.3 million or when the lifetime costs of trisomies 13 and 18 were below $704,000. However, contingent NIPT remained less costly than MSS over the full range of lifetime costs that was covered in the one-way analysis. This remained true even when the lifetime cost of trisomies 13 and 18 were excluded from the analysis, due to the low birth prevalence of these trisomies. Therefore, our conclusions are robust even when taking into account uncertainty surrounding the lifetime cost estimates, particularly those of trisomies 13 and 18.

For this reason, assumptions about trisomies 13 and 18 have relatively little impact on the analysis.

Our study also has several strengths. Our analysis included trisomies 13 and 18, which have only been included in one previous analysis.[8] Although other studies have investigated contingent policies, we identified the optimal risk cutoffs and were able to compare the cost effectiveness of an optimal contingent policy against the cost effectiveness of universal NIPT and MSS. Our analysis shows the potential cost savings of contingent NIPT. We included multiple cost perspectives. We analyzed cost effectiveness from a broad allocative standpoint (societal perspective), as well as from narrower perspectives that would be relevant to government and payer decision makers. Finally, our results were robust. We found that contingent NIPT was less costly than MSS over a wide range of costs and probabilities used in our analysis.

## Conclusion

From a societal perspective, universal NIPT is a cost-effective alternative to MSS and contingent NIPT. When viewed from government or payer perspectives, contingent NIPT is cost-effective relative to MSS but is both less costly and less effective than universal NIPT. In these cases, the choice of policy depends on the willingness to pay for additional detections. Adopting universal NIPT would cost $203,088 for each additional case detected from a government perspective and $263,922 for each additional case detected from a payer perspective.

## Supporting Information

S1 Text Explanation of Lifetime Cost Estimates. (DOCX)
