# Supplementary material for: A Cost-Effectiveness Analysis of First Trimester Non-Invasive Prenatal Screening for Fetal Trisomies in the United States
Source: PLoS One. 2015 Jul 2;10(7):e0131402. doi: 10.1371/journal.pone.0131402 (PMC4489811; doi:10.1371/journal.pone.0131402)
Supplement: S1 Text — (DOCX) [file pone.0131402.s001.docx]

# S1 Explanation of-lifetime cost estimates

Lifetime costs were estimated using a standard cost of illness methodology using age-specific annual costs and contributions to productivity with survival data.[^1^](#_ENREF_1) Lifetime costs represent the difference in expected costs between a trisomy birth and average birth. Lifetime costs include direct medical and educational costs and the indirect costs due to lost productivity.

To update the costs of trisomy 21, we used more recent survival data to reflect longer life expectancies. The lifetime costs of trisomies 13 and 18 were estimated using the cost data provided by Waitzman for trisomy 21 and using trisomies 13 and 18 survival data. Trisomies 13 and 18 have similar one-year survival rates.[^2^](#_ENREF_2) In the absence of survival data from trisomy 13 beyond one year, we assumed survival beyond one year was similar to that of trisomy 18.[^3^](#_ENREF_3) Therefore, the same survival curve was used for trisomies 18 and 13.

Costs beyond one year were discounted at an annual rate of 3% as recommended by cost-effectiveness guidelines.[^4^](#_ENREF_4)^,^[^5^](#_ENREF_5) Lifetime cost estimates were inflated to 2013 dollars. We adjusted the medical portion of lifetime costs using the health care component of the personal consumption expenditure index.[^6^](#_ENREF_6) We adjusted the non-medical direct and indirect portions of lifetime costs using the employment cost index for civilian workers.[^7^](#_ENREF_7)

The estimated direct costs were $427,577 for trisomy 21 and $37,971 for trisomies 13 and 18. Because few trisomy 13 or 18 live births survived beyond one year, the bulk of direct costs were comprised of medical costs within the first year. The estimated indirect costs due to lost productivity were $1,069,195 for trisomy 21 and $1,363,877 for trisomies 13 and 18. These costs represent the difference in estimated productivity contributions between an average and trisomy birth. The indirect costs are larger for trisomies 13 and 18 compared to trisomy 21 due to the short life expectancy that precludes productivity contributions for trisomies 13 and 18.

The direct medical portion of the lifetime costs of trisomies 13 and 18 were the greatest source of uncertainty in these estimates. We are not aware of any study that examines annual expenditures for trisomies 13 and 18. However, one study found the average hospitalization for trisomies 13 and 18 infants resulted in hospital charges of $30,000-$39,000 (2003 dollars).[^8^](#_ENREF_8) Although a direct comparison cannot be made between our medical cost estimates, these figures suggest our estimates may have understated these costs.

**Citations**

1. Waitzman NJ, Scheffler RM, Romano PS. *The cost of birth defects: estimates of the value of prevention.* University Press of America; 1996.

2. Vendola C, Canfield M, Daiger SP, Gambello M, Hashmi SS, King T, et al. Survival of Texas infants born with trisomies 21, 18, and 13. *American journal of medical genetics. Part A*;152a(2):360-366.

3. Niedrist D, Riegel M, Achermann J, Schinzel A. Survival with trisomy 18--data from Switzerland. *American journal of medical genetics. Part A*;140(9):952-959.

4. Gold M, Siegel J, Russell L, Weinstein M. *Cost-Effectiveness in Health and Medicine.* USA: Oxford University Press; 1996.

5. Drummond M, Sculpher M, Torrance G, O'Brien B, Stoddart G. *Methods for the Economic Evaluation of Health Care Programmes.* 3 ed: Oxford University Press, Incorporated; 2005.

6. Personal Consumption Expenditures Index. <http://www.bea.gov/>. Accessed April 15, 2014.

7. Employment Cost Index. <http://www.bls.gov>. Accessed April 15, 2014.

8. Hospital stays, hospital charges, and in-hospital deaths among infants with selected birth defects--United States, 2003. *MMWR Morb Mortal Wkly Rep*;56(2):25-29.
